# Defining the index trauma in post-traumatic stress disorder patients with multiple trauma exposure: impact on severity scores and treatment effects of using worst single incident versus multiple traumatic events

**DOI:** 10.1080/20008198.2018.1486124

**Published:** 2018-07-09

**Authors:** Kathlen Priebe, Nikolaus Kleindienst, Andrea Schropp, Anne Dyer, Antje Krüger-Gottschalk, Christian Schmahl, Regina Steil, Martin Bohus

**Affiliations:** a Institute for Psychiatric and Psychosomatic Psychotherapy, Central Institute of Mental Health, Medical Faculty Mannheim, Heidelberg University, Mannheim, Germany; b Department of Psychiatry and Psychotherapy, Charité – Universitätsmedizin Berlin, Berlin, Germany; c Institute for Neuropsychology and Clinical Psychology, Central Institute of Mental Health, Medical Faculty Mannheim, Heidelberg University, Mannheim, Germany; d Department of Clinical Psychology and Psychotherapy, University of Münster, Münster, Germany; e Department of Health, Antwerp University, Antwerp, Belgium; f Department of Psychosomatic Medicine and Psychotherapy, Central Institute of Mental Health, Medical Faculty Mannheim, Heidelberg University, Mannheim, Germany; g Department of Psychiatry, Schulich School of Medicine and Dentistry, Western University, London, Ontario, Canada; h Department of Psychology and Sports Sciences, Goethe University Frankfurt, Frankfurt, Germany

**Keywords:** PTSD, Criterion A, index trauma, cumulative trauma, multiple traumatization, TEPT, Criterio A, Trauma índice, trauma acumulado, traumatización múltiple, PTSD, 标准A, 指标创伤, 累积创伤, 多重创伤, • This study demonstrates the importance of taking the effects of multiple traumatic events into account when assessing PTSD.• We found higher PTSD severity scores and less improvement after trauma-focused psychotherapy when the index trauma included multiple distinct traumatic events compared to when the index trauma was defined as the worst single incident.• A broader definition of index trauma may provide a more comprehensive view on PTSD severity and treatment effects.

## Abstract

**Background:** A diagnosis of post-traumatic stress disorder (PTSD) requires the identification of one or more traumatic events, designated the index trauma, which serves as the basis for assessment of severity of PTSD. In patients who have experienced more than one traumatic event, severity may depend on the exact definition of the index trauma. Defining the index trauma as the worst single incident may result in PTSD severity scores that differ from what would be seen if the index trauma included multiple events.

**Objective:** This study aimed to investigate the impact of the definition of the index trauma on PTSD baseline severity scores and treatment outcome.

**Method:** A planned secondary analysis was performed on data from a subset (*N *= 58) of patients enrolled in a trial evaluating the efficacy of a 12 week residential dialectical behavioural therapy programme for PTSD related to childhood abuse (DBT-PTSD). Assessments of the severity of PTSD were conducted at admission, at the end of the 12 week treatment period, and at 6 and 12 weeks post-treatment, using the Clinician-Administered PTSD Scale. The index trauma was defined with respect to both the worst single incident and up to three qualitatively distinct traumatic events.

**Results:** When the index trauma included multiple traumas, PTSD severity scores were significantly higher and improvements from pre- to post-treatment were significantly lower than when the index trauma was defined as the worst single incident.

**Conclusions:** In patients with PTSD who have experienced multiple traumas, defining the index trauma as the worst single incident may miss some aspects of clinically relevant symptomatology, thereby leading to a possibly biased interpretation of treatment effects. In DBT-PTSD, treatment effects were lower when the index trauma included multiple traumatic events. More research is needed to determine the impact of the various index trauma definitions on the evaluation of other trauma-focused treatments.

## Abstract (es)

**Antecedentes**: Para diagnosticar un trastorno de estrés postraumático (TEPT) se requiere la identificación de uno o más eventos traumáticos. La designación del trauma índice sirve para evaluar la severidad del TEPT. En pacientes que han experimentado más de un evento traumático, la severidad podría depender de la definición exacta que se le otorgue al trauma índice. Definir el trauma índice como el peor incidente podría resultar en puntajes de severidad diferentes a los obtenidos si el trauma índice incluyera o comprendiera eventos múltiples.

**Objetivo**: Este estudio investiga el impacto de la definición del trauma índice sobre los puntajes de severidad basal de TEPT y los resultados del tratamiento.

**Método**: Se realizó un análisis secundario planificado sobre los datos de una muestra (N = 58) de pacientes reclutados para un ensayo que evaluaba la eficacia de un programa residencial DBT-TEPT de 12 semanas para TEPT relacionado a abuso infantil. Se evaluó la severidad del TEPT usando la escala de TEPT Administrada por el clínico al inicio, al final del periodo de 12 semanas de tratamiento, y a las 6 y 12 semanas posteriores al tratamiento. El trauma índice se definió tanto para el peor incidente como para hasta tres eventos cualitativamente distintos.

**Resultados**: Cuando el trauma índice incluye múltiples traumas, los puntajes de severidad de TEPT fueron significativamente más altos y la mejoría posterior al tratamiento fue significativamente más baja comparado a cuando el trauma índice era definido solamente con el peor incidente.

**Conclusiones**: En pacientes con TEPT que han experimentado múltiples traumas, definir el trauma índice con el peor incidente puede pasar por alto algunos aspectos de la sintomatología clínicamente relevantes, conduciendo a posibles interpretaciones sesgadas de los efectos del tratamiento. En DBT-TEPT, los efectos del tratamiento fueron menores cuando el trauma índice incluyó eventos traumáticos múltiples. Se requiere mayor investigación para determinar el impacto de las diversas definiciones de trauma índice sobre la evaluación de resultados de otros tratamientos focalizados en trauma.

## Abstract (zh)

**背景**：诊断创伤后应激障碍（PTSD）需要确定一项或多项创伤事件，称为指标创伤，作为评估PTSD严重程度的基础。 在经历多于一次创伤性事件的患者中，严重程度可能取决于指标创伤的确切定义。 将指数创伤定义为最差的单一事件可能导致PTSD严重程度分数与如果让指数创伤包括多个事件之后的 PTSD 分数不同。

**目的**：本研究调查指数创伤的定义对PTSD基线严重度评分和治疗结果的影响。

**方法**：对纳入试验的患者（N = 58）的数据按计划进行二次分析，数据来自评估一个12周住院DBT-PTSD计划对与儿童期虐待有关的PTSD疗效的项目。 PTSD严重程度的评估在入院时，12周治疗结束时和治疗后6周和12周时使用临床医生管理的PTSD量表进行。 指数创伤被定义为最严重的单一事件和多达三个性质不同的创伤性事件。

**结果**：当指数创伤包括多次创伤时，PTSD严重度评分显著较高，治疗前后的改善效果显著低于当指数创伤被定义为最差的单一事件。

**结论**：PTSD患者经历过多次创伤，定义指数创伤是最差的单一事件可能会错过临床相关症状的某些方面，从而导致对治疗效果可能存在偏倚的解释。 在DBT-PTSD中，当指数损伤包括多次创伤事件时，治疗效果较低。 需要更多的研究来确定各种指数创伤定义对其他创伤治疗评估的影响。

## 1. Introduction

Post-traumatic stress disorder (PTSD) first appeared in the Diagnostic and Statistical Manual of Mental Disorders in the third edition (DSM-III) (American Psychiatric Association, 1980). A diagnosis of PTSD requires exposure to a traumatic event that is referred to as Criterion A. One or more traumatic events, designated the index trauma, must be identified, and only symptoms related to the index trauma are considered in the diagnosis and evaluation of the severity of PTSD. However, the question of which events qualify as traumatic events has been the subject of controversial debate (Weathers & Keane, 2007), and each edition of the DSM since the third has seen a modification of Criterion A. Less attention has been paid to how the index trauma should be defined in patients with a history of multiple traumatic events. Epidemiological studies have consistently found that exposure to multiple traumatic events is quite common (Kessler, Sonnega, Bromet, Hughes, & Nelson, 1995; Kilpatrick et al., 2013). In DSM-IV (American Psychiatric Association, 2000), the definition of Criterion A used the wording ‘has been exposed to a traumatic event’ (p. 427), which DSM-5 (American Psychiatric Association, 2013) notably modified to ‘traumatic event(s)’ (p. 271). However, it does not specify what is meant by ‘event(s)’.

Assessment of PTSD usually begins with presenting individuals with a list of event types, such as the Life Events Checklist (LEC) (Weathers,  Blake et al., 2013), in which they are asked to indicate the events that they have experienced over the course of their life. In epidemiological studies, participants who have experienced multiple traumatic events are typically asked to identify the worst trauma, defined as the currently most distressing event (e.g. Kessler et al., 1995). In the more recent World Mental Health Survey, PTSD was additionally assessed in relation to a traumatic event randomly selected from among those endorsed by the participants (Kessler et al., 2017).

Diagnostic interviews and questionnaires that are used for assessing PTSD diagnosis and severity differ in their exact definition of the index trauma. Two of the most widely used interviews are the Clinician-Administered PTSD Scale (CAPS) and the Posttraumatic Stress Disorder Symptom Scale Interview (PSSI). The CAPS for DSM-IV (Blake et al., 1995) allows the assessment of post-traumatic symptoms to be based on up to three different traumatic events, whereas the CAPS for DSM-5 (CAPS-5) (Weathers, Blake et al., 2013, p. 2) defines an index event as either the worst single incident (e.g. ‘the accident’) or multiple but closely related incidents (e.g. ‘the worst parts of your combat experiences’). In the PSSI for DSM-5 (PSSI-5) (Foa, McLean, Zang, Zhong, Rauch et al., 2016), if respondents have experienced more than one traumatic event, they are asked to select ‘the traumatic event that is currently most distressing’ (p. 1160). Accordingly, in patients with exposure to multiple traumatic events, the index trauma chosen for the diagnosis and severity of PTSD differs as a function of the instrument and may represent a single incident (e.g. a traffic accident), or multiple closely related incidents (e.g. multiple incidents of prolonged child abuse), or exposure to multiple qualitatively distinct events (e.g. a traffic accident plus prolonged child abuse). Also, both the CAPS and the PSSI, as well as other instruments, have been used in a wide variety of ways for specific research purposes, for example, with reference to all upsetting events including those not meeting Criterion A, without reference to specific traumatic events or with reference to specific traumatic events. Despite the various options in the literature for handling multiple traumas (e.g. worst event, randomly selected event, up to three events), empirical research regarding the impact of the exact index trauma definition on the assessed rates and severity of PTSD is scant.

Research has shown that the rates and symptom presentation of PTSD differ across types of trauma, with interpersonal trauma often being associated with a higher probability of PTSD and more severe symptomatology than non-interpersonal trauma (e.g. Kessler et al., 2017, 1995; Smith, Summers, Dillon, & Cougle, 2016). Besides the type of trauma, numerous studies have found that cumulative trauma, mostly defined as the number of different trauma types, is associated with greater PTSD risk and symptom severity (Green et al., 2000; Karam et al., 2014; Wilker et al., 2015). Simpson, Comtois, Moore, and Kaysen (2011) found that the prevalence of PTSD increased from 53.7% to 67.2% when patients were asked to rate their symptoms for their complete trauma history instead of for only their worst event. Similarly, Breslau, Davis, Peterson, and Schultz (1997) found in a sample of women with PTSD that 16.0% of the cases were detected only when ‘the worst event’ was extended to include other traumatic events. Beals et al. (2013) studied the prevalence of PTSD in two Native American populations. When assessment was related to the worst event prevalence, estimates ranged from 5.9% to 14.8%, compared to prevalence rates between 8.9% and 19.5% when the assessment was based on three worst traumatic events. In a study with a non-clinical sample, Elhai et al. (2009) tested whether PTSD’s factor structure differed when based on a worst single incident versus the complete trauma history. No differences in symptom constellation or severity were evident across conditions; however, owing to the non-clinical nature of this sample, it may be difficult to generalize the results to clinical samples.

According to Stein, Wilmot, and Solomon (2016), for an individual with multiple traumas, each trauma may result in different symptoms which only in combination will fulfil the criteria for PTSD; alternatively, several traumatic events may each be associated with all diagnostic criteria and lead to overlapping PTSD. The authors tentatively termed these conditions ‘cumulative’ versus ‘multiple’ PTSD. These assumptions were supported by the results of a large cross-national, population-based survey (Karam et al., 2014), which found not only that respondents with PTSD who had been exposed to multiple traumas had a higher probability of PTSD, but also that nearly 20% of these respondents attributed their current PTSD symptoms to more than one traumatic event. The practice of choosing a single traumatic event in individuals with multiple trauma exposure does not account for these cumulative effects of multiple traumas.

To determine which definitions of index trauma are currently being used in psychotherapy research, we searched the literature for studies on the treatment of patients with a diagnosis of PTSD after repeated childhood abuse. The most recent meta-analysis on this topic (Ehring et al., 2014) included 16 randomized controlled trials (RCTs), and we identified one more (Jung & Steil, 2013). Details of these 17 studies are presented in Supplementary Table 1. Thirteen of the trials gave no definition of the index trauma. Of the four studies that did specify what definition they were using, all used a narrow definition, such as ‘worst event’ (Resick et al., 2008) or ‘a CSA [childhood sexual abuse] experience’ (McDonagh et al., 2005). Importantly, we did not find any study in which more than one definition of the index trauma was used to evaluate its treatment outcomes. Our conclusion from the literature search was that the impact of the definition of the index trauma on treatment effects remains unclear.

**Table 1.** Baseline characteristics.

	DBT-PTSD (*n* = 29)	TAU-WL (*n* = 29)	*p*
Age (years)	35.66 (10.82)	36.34 (8.32)	0.75^a^
Years of education	13.00 (2.77)	12.17 (2.00)	0.33^a^
Number of current Axis I disorders	2.97 (1.15)	2.93 (1.00)	0.80^a^
Score on BDI-II	38.03 (9.76)	41.00 (9.07)	0.24^a^
Number of BPD criteria met	4.21 (1.63)	4.45 (1.92)	0.61^a^

Data are expressed as mean (*SD*).

^a^ Mann–Whitney *U* test.

DBT-PTSD, dialectical behavioural therapy for post-traumatic stress disorder; TAU-WL, treatment-as-usual waiting list; BDI-II, Beck Depression Inventory-II; BPD, borderline personality disorder.

It seems plausible that the definition of the index trauma is more relevant when the PTSD symptoms being evaluated are closely related to the traumatic event, such as intrusions, than when they are more general, such as hypervigilance. Bovin and Weathers (2012) grouped the 17 symptoms of DSM-IV PTSD (American Psychiatric Association, 2000) into two clusters: eight symptoms that are inherently linked to the trauma, and nine that are not. The first cluster comprises five symptoms of re-experiencing (Criterion B), the two avoiding stimuli (Criterion C), and amnesia (formerly Criterion C), and are referred to as ‘trauma-related symptoms’. The other nine symptoms, which are only functionally related to the specific index trauma, are associated with numbing of responsiveness (formerly Criterion C) and symptoms of hyperarousal (formerly Criterion D), and are referred to as ‘non-specific symptoms’. If trauma-related symptoms can be clearly linked to a particular traumatic event and non-specific symptoms cannot, it seems likely that different definitions of index trauma will have an impact on trauma-related but not on non-specific symptoms.

The objective of the present study was to investigate the impact of using two different definitions of index trauma on both the baseline PTSD severity score and PTSD treatment outcome. To this end, we carried out a planned secondary analysis of a previously published RCT (Bohus et al., 2013), which compared the treatment effects of a 12 week residential programme of dialectical behavioural therapy for post-traumatic stress disorder (DBT-PTSD) to a treatment-as-usual waiting list (TAU-WL). In that study, large between-group effect sizes were found when the index trauma was defined as the worst single CSA incident.

The present study compared the results using both the above definition and a definition of multiple traumas, where the latter involved up to three qualitatively distinct events. Three research questions were addressed: (1) Do the PTSD severity scores differ if the assessment is focused on symptoms related to the worst single incident or on multiple traumas? (2) Do these different definitions affect how much improvement is seen in PTSD severity scores? and (3) Do improvements in PTSD severity scores regarding ‘trauma-related’ symptoms versus ‘non-specific’ symptoms differ when assessment is based on one or the other of these definitions? Based on current evidence that links multiple trauma exposure with increased PTSD rates and severity, we hypothesized greater PTSD severity, especially of trauma-related symptoms, when PTSD was assessed in relation to multiple events rather than in relation to the worst single incident. DBT-PTSD is a trauma-focused treatment that includes interventions from DBT as well as trauma-focused cognitive and exposure-based interventions. Sessions on trauma-related cognitions and acceptance of trauma-related facts take all traumatic events into account, while the imaginal exposure is conducted in relation to the currently most distressing incident. Accordingly, we hypothesized greater improvement in PTSD symptoms related to this currently most distressing incident (worst single incident) than in PTSD symptoms related to multiple events.

## 2. Method

### 2.1. Participants

Participants in the RCT were females aged 17–65 years who met the inclusion criteria of a DSM-IV diagnosis of PTSD related to sexual abuse before the age of 18, plus at least one of the following additional diagnoses: current major depressive disorder, eating disorder, substance abuse, or at least four DSM-IV criteria of borderline personality disorder (BPD). CSA had to be the currently most distressing trauma. Exclusion criteria included a lifetime diagnosis of schizophrenia, body mass index < 16.5 kg/m^2^, current substance dependence, intellectual disability, and medical conditions that contradicted the exposure protocol.

All participants provided written informed consent. Approval was obtained from the ethics committee of the Medical Faculty Mannheim at Heidelberg University (trial registration: ClinicalTrials.gov, number NCT00481000).

### 2.2. Procedure

DBT-PTSD is a 12 week residential treatment programme designed for patients with PTSD with severe emotion dysregulation. Patients receive twice-weekly 45 minute sessions of individual treatment (a total of 23 sessions over the 12 weeks) plus weekly group treatment. DBT-PTSD is based on the principles and methods of DBT (Linehan, 1993), and integrates trauma-focused cognitive and exposure-based interventions as described by Ehlers and Clark (2000) and Foa, Hembree, and Rothbaum (2007). The programme is divided into three phases. In Phase 1 (weeks 1–4), patients identify their individual avoidance strategies on a cognitive, emotional, and behavioural level, and, with the help of individualized behavioural analysis, learn to use specific DBT skills to control crisis-generating behaviours and dissociative features. In Phase 2 (weeks 5–10), the focus is on trauma-specific cognitive and exposure-based interventions. Exposure is usually addressed over approximately seven individual sessions, and focuses on the currently most distressing CSA incident. In the present study, we used that incident as the ‘worst single incident’ for one of the definitions of index trauma. Phase 3 (weeks 11 and 12) aims to improve radical acceptance of trauma-related and biographical facts. For details of the DBT-PTSD programme, see Bohus et al. (2013) and Steil, Dyer, Priebe, Kleindienst, and Bohus (2011).

The trial was conducted at a single residential treatment centre. A total of 74 participants were randomly assigned to either the treatment group (*n *= 36), in which all patients received DBT-PTSD, or the TAU-WL group (*n *= 38), in which they could receive any form of treatment other than DBT-PTSD. The total duration of participation in the study was 24 weeks, comprising 12 weeks of treatment and 12 weeks of follow-up. Assessments were conducted by trained and experienced clinicians at admission (week 0; t1), discharge (week 12; t2), 6 week follow-up (week 18; t3), and 12 week follow-up (week 24; t4). The raters were blinded to study treatment. Following the last assessment, participants who had been assigned to the TAU-WL group were offered DBT-PTSD treatment if they wished.

### 2.3. Measures

The following instruments were administered at baseline: the Structured Clinical Interview for DSM-IV Axis I Disorders (SCID-I) (First, Spitzer, Gibbon, & Williams, 1996) to diagnose Axis I disorders; the International Personality Disorder Examination (IPDE) (Loranger et al., 1994) to determine the severity of BPD, and the Beck Depression Inventory-II (BDI-II) (Beck, Steer, & Brown, 1996) to assess severity of depressive symptoms. Exposure to different types of traumatic events was assessed using the LEC (Blake et al., 1995) and the Posttraumatic Stress Diagnostic Scale (PDS) (Foa, 1995). The merged scale contained 21 different types of traumatic event. More details of the merged list are provided in Supplementary Table 2.

**Table 2.** Overview of trauma history.

	DBT-PTSD (*n* = 29)	TAU-WL (*n* = 29)	*p*
Number of different trauma types^a^	7.00 (3.58)	5.77 (2.37)	0.31^b^
Range	2–16	2–11	
			
*Index trauma definition: Multiple traumas – assessed (max. 3)*			
Total	2.79 (0.56)	2.83 (0.47)	0.96^b^
One traumatic event (%)	6.9	3.4	
Two traumatic events (%)	6.9	10.3	
Three traumatic events (%)	86.2	86.2	
CSA with all incidents			
Age at start of abuse (years)	7.45 (3.93)	8.30 (4.37)	0.49^b^
Abused by family member (%)	79.3	72.4	0.76^d^
Duration			0.99^c^
Single incident (%)	17.2	11.1	
< 5 years (%)	31.0	33.3	
5–10 years (%)	44.8	40.8	
> 10 years (%)	6.9	14.8	
Frequency			0.86^c^
Single incident or seldom (%)	25.0	18.5	
From several times a month to weekly (%)	35.7	25.9	
From several times a week to daily (%)	39.3	55.6	
With penetration (%)	89.3	77.8	0.30^d^
Additional traumatic events			
Another CSA (different abuser) (%)	55.2	60.7	0.79^d^
Child physical abuse (%)	53.6	53.6	0.99^d^
Adult sexual assault (%)	20.7	25.0	0.76^d^
Adult physical violence (%)	17.2	10.7	0.71^d^
*Index trauma definition: Worst single incident*			
Age at time of incident (years)	9.32 (3.71)	10.73 (3.71)	0.17^b^
With penetration (%)	78.6	68.0	0.53^d^

Data are expressed as mean (*SD*) or as number in %.

^a^ Different trauma types are listed in Supplementary Table 2. ^b^ Mann–Whitney *U* test. ^c^ Kolomogorov–Smirnov test. ^d^ Fisher’s exact test.

DBT-PTSD, dialectical behavioural therapy for post-traumatic stress disorder; TAU-WL, treatment-as-usual waiting list; CAPS, Clinician-Administered PTSD Scale; CSA, childhood sexual abuse.

The CAPS (Blake et al., 1995) was used to determine PTSD diagnosis and severity throughout the study, rating the 17 PTSD symptoms according to DSM-IV. The CAPS for DSM-IV allows the separate quantification of the frequency and intensity of each symptom using five-point scales. Frequency and intensity ratings are summed for each symptom to obtain a severity score and across symptoms to obtain an overall severity of PTSD. In a series of studies, the CAPS has demonstrated excellent psychometric properties (Blake et al., 1995). At the start of the RCT, the CAPS was used only to assess symptoms in relation to the worst CSA incident; however, as several patients revealed that they attributed their symptoms to more than one trauma, it was subsequently used to assess symptoms in relation to multiple traumas as well. Accordingly, PTSD symptom severity was assessed in relation to two definitions of index trauma: ‘multiple traumas’ and ‘worst single incident’. In correspondence with the CAPS for DSM-IV, the assessment of ‘multiple traumas’ included up to three distinct traumatic events, and was defined as all of the experiences of the most distressing CSA trauma, as well as up to two other qualitatively distinct traumatic events, including all their corresponding single incidents. Event types did not have to vary. For example, CSA conducted by the father lasting 5 years was counted as one CSA, and rape at age 15 by a stranger as another CSA. The ‘worst single incident’ was defined as the currently most distressing single CSA incident. Before the first CAPS assessment, the patients were given the LEC to assess exposure to different traumatic events during their lifetime. Subsequently, the three most distressing traumatic events (CSA and two other events) as well as the most distressing single CSA incident were determined. During the CAPS assessment, each symptom was assessed in relation to multiple traumas first, and then the symptom was assessed again in relation to the worst single incident. The severity of PTSD was calculated as the total severity score over criteria B, C, and D, and ranged from 0 to 136. As the symptom groups (i.e. trauma-related and non-specific) contained different numbers of symptoms, the mean of all related symptoms was calculated. The obtained mean scores for each symptom group ranged from 0 to 4.

### 2.4. Data analysis

To test whether the level and change in PTSD severity depend on the exact definition of the index trauma (i.e. ‘worst single incident’ vs ‘multiple traumas’), the hierarchical linear model approach from the main publication (Bohus et al., 2013) was used. In a first step, the random-slope random-intercept model with predictors for group (coded as 1 = DBT-PTSD, 0 = TAU-WL), time (in weeks), and the interaction of group × time was used to model PTSD severity scores (Model 1). For the purpose of the present study, this model was extended by adding index trauma (coded as 1 = worst single incident, 0 = multiple traumas) as an independent variable (Model 2). The resulting model was further extended by an interaction term (index trauma × group × time) to test whether different index trauma definitions differentially affect the change over time across the two treatment groups (Model 3). Finally, significant deviation from linearity in the DBT-PTSD group at week 12 was addressed by adding the respective indicator function (1 = DBT-PTSD group at week 12) to the model 3 resulting in Model 4. Parameters were estimated from full maximum likelihood estimators (Luke, 2004). The nested models 1–4 were sequentially compared with likelihood ratio tests. All cases where PTSD symptom severity scores were assessed in relation to both index trauma definitions were evaluated, even if there were data points missing. To account for systematic bias from study non-completers, separate analyses with and without non-completers were conducted (Little et al., 2012).

Improvements and differences within the symptom groups between different index trauma definitions were tested using the Wilcoxon signed rank test. To quantify changes, Hedges’ *g* effect size was used. Tests were considered to be statistically significant if a *p* value of 0.05 or smaller was reached (two-tailed). Calculations were conducted with SAS™ version 9.4 and SPSS version 21.

## 3. Results

### 3.1. Patient flow

The main analysis (hierarchical linear model) included all randomized patients who had provided at least one measurement of PTSD severity in relation to the worst single incident plus at least one measurement in relation to multiple traumas at any assessment time. This means that patients were also included when they had provided one measurement in relation to the worst single incident at a certain assessment time, and one measurement in relation to multiple traumas at another assessment time. The two different definitions of index trauma were assessed in a subsample of 58 participants, 29 from each group. Of these, two patients in the DBT-PTSD group and three in the TAU-WL group dropped out before week 12 (t2) and declined further assessment (treatment non-completers). At week 24 (t4), an additional four patients (three in the DBT-PTSD group, one in the TAU-WL group) did not complete the follow-up assessments (study non-completers). Hence, at week 24, data were missing for a total of nine patients.

In the comparisons of CAPS scores for the two definitions of index trauma, we included only patients for whom both measurements were available for the same assessment time-point. In the DBT-PTSD group, there were 24 such patients available at baseline, 22 at end of treatment, 21 at the 6 week follow-up, and 24 at the 12 week follow-up, while in the TAU-WL group, these numbers were 23, 22, 22, and 25, respectively. Further information in regard to patient flow is presented in Supplementary Figure 1.

### 3.2. Participant characteristics

Baseline sociodemographic and clinical characteristics are presented in Table 1. Mean age was 35.66 years (*SD *= 10.82, range = 19–52) in the DBT-PTSD group and 36.34 years (*SD *= 8.32, range = 20–52) in the TAU-WL group. The mean totals of current Axis I disorders were 2.97 (*SD *= 1.15) and 2.93 (*SD *= 1.00), respectively. There were no significant differences in any baseline sociodemographic or clinical characteristics between the treatment groups or between study completers and study non-completers.

### 3.3. Trauma history

Overall, participants reported having been exposed to a mean of 6.37 (*SD *= 3.06) different types of trauma. When participants were asked to select up to three currently distressing traumatic events including the CSA, almost all of them (94.8%) chose more than one traumatic event. On average, participants chose 2.81 events (*SD *= 0.51). Typically, the CSA had started at a mean age of 7.86 years (*SD *= 4.13), had been perpetrated by a family member (75.9%), and had included penetration (83.6%). Often, the abuse had lasted for more than 5 years (53.6%), and had occurred monthly or more often (78.2%). The single worst CSA incident was reported to have occurred at a mean age of 10.00 years (*SD *= 3.74), and included penetration in 73.3% of cases. Most of the additional traumatic events were other occurrences of sexual violence (57.9%), followed by physical abuse (53.6%). Additional characteristics of trauma history are presented in Table 2.

There were no significant differences in terms of participants’ characteristics in regard to trauma exposure, CSA characteristics, and CAPS scores between the DBT-PTSD group and the TAU-WL group or between study completers and study non-completers.

### 3.4. CAPS total severity score overview

**Figure 1.**
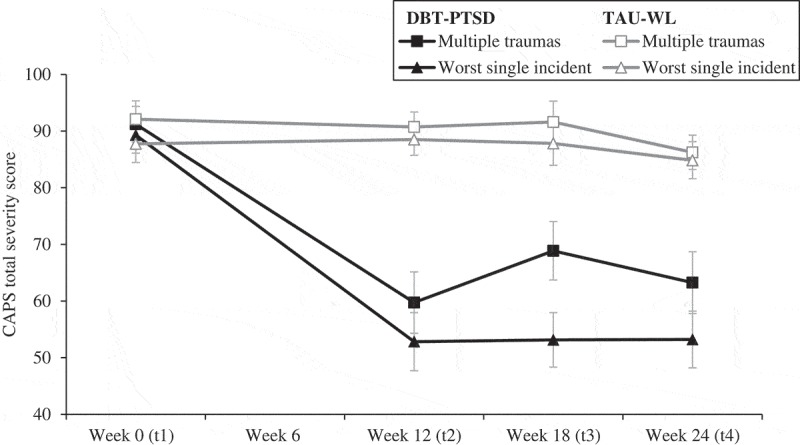
Change in total Clinician-Administered PTSD Scale (CAPS) severity score displayed as group means with standard errors in dependency of different index definitions (worst single incident vs multiple traumas) over time for the dialectical behavioural therapy for post-traumatic stress disorder (DBT-PTSD) and treatment-as-usual waiting list (TAU-WL) groups.

Results of the CAPS total severity score are summarized in Table 3 and graphically displayed in Figure 1; and the mean CAPS scores for Criteria B, C, and D are presented in Supplementary Table 3. PTSD severity scores were always lower when the assessment was conducted in relation to the worst single incident as compared to multiple traumas, with the difference reaching significance at all but one time-point (comparison at week 24 in the TAU-WL group).

**Table 3.** Clinician-Administered PTSD Scale (CAPS) total severity scores.

	DBT-PTSD				TAU-WL			
	*n*	Worst single incident	Multiple traumas	*p*^a^	*n*	Worst single incident	Multiple traumas	*p*
Week 0 (admission, t1)	24	89.29 (15.60)	91.21 (15.39)	.015	23	87.74 (15.73)	92.09 (15.48)	.002
Week 12 (discharge, t2)	22	52.82 (24.04)	59.73 (25.36)	< .001	22	88.50 (13.02)	90.73 (12.38)	.004
Week 18 (6 week follow-up, t3)	21	53.14 (22.01)	68.86 (23.65)	< .001	22	87.82 (18.09)	91.59 (17.33)	.009
Week 24 (12 week follow-up, t4)	24	53.21 (24.52)	63.25 (26.75)	< .001	25	84.84 (16.37)	86.24 (15.24)	.104
Hedges’ *g* (within-group; t1–t4)		1.73	1.26			0.18	0.37	

Data are expressed as mean (*SD*).

^a^ Wilcoxon signed rank test.

DBT-PTSD, dialectical behavioural therapy for post-traumatic stress disorder; TAU-WL, treatment-as-usual waiting list.

**Figure 2.**
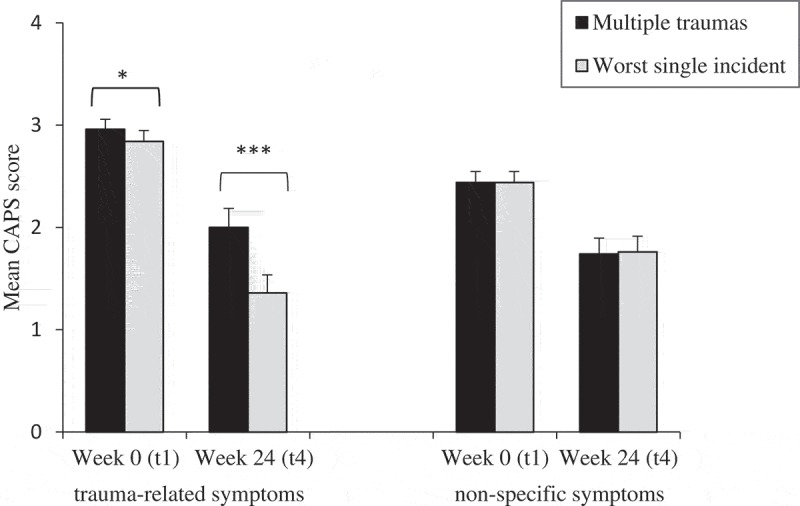
Mean Clinician-Administered PTSD Scale (CAPS) score for week 0 (t1) and week 24 (t4) with standard errors within trauma-related and non-specific symptoms presented as group means in the dialectical behavioural therapy for post-traumatic stress disorder (DBT-PTSD) group in relation to different index trauma definitions (multiple traumas vs worst single incident). *p ≤ .05, ***p ≤ .001 (Wilcoxon signed rank test).

### 3.5. Treatment effects

The mean change in the CAPS scores was larger in the DBT-PTSD group than in the TAU-WL group when the index trauma was defined as the worst single incident (36.08 vs 2.90) as well as when the index trauma included multiple traumas (27.96 vs 5.85). As shown in Table 4, Model 1 indicated an additional weekly decline of −1.23 points on the total CAPS severity score (*SE *= 0.24, *p *< .001) in the DBT-PTSD group compared to the TAU-WL group, independent of the index trauma definition. Adding index trauma as a predictor to the basic hierarchical linear model significantly increased the model fit (*χ*^2^(1, *n *= 378) 3167.64 − 3153.25 = 14.39, *p *< .001). With a point estimate of −5.01 (*SE *= 1.30, *p *< .001), the predictor for index trauma was significant, indicating that the worst single incident compared to multiple traumas was related to lower CAPS total scores during the observation period. Further analyses (Model 3) revealed a significant three-way interaction of index trauma × time × group, indicating that the steeper decline in the CAPS total scores referring to the worst single incident (vs multiple traumas) was more pronounced in the DBT-PTSD group than in the TAU-WL group. Inclusion of the post-treatment × group term in the final model (Model 4) led to a significantly better fit (*χ*^2^(1, *n *= 378) 3143.95 − 3074.76 = 69.19, *p *< .001).

**Table 4.** Hierarchical linear models.

	Model 1	Model 2	Model 3	Model 4
Intercept	88.11***(2.79)	91.22***(2.88)	90.04***(2.89)	89.95*** (2.84)
Time	−0.12 (0.17)	−0.10 (0.17)	−0.11 (0.17)	−0.11 (0.17)
Group	−5.71 (3.94)	−5.52 (3.99)	−5.62 (3.97)	−1.11 (3.97)
Time × Group	−1.23*** (0.24)	−1.24*** (0.25)	−1.02*** (0.26)	−1.02*** (0.25)
Index trauma		−5.01*** (1.30)	−2.26 (1.56)	−2.23 (1.38)
Index trauma × Time × Group			−0.43** (0.14)	−0.42*** (0.12)
Post-treatment × Group				−16.66*** (1.89)
Parameters to be estimated	8	9	10	11
−2*log likelihood	3167.64	3153.25	3143.95	3074.76
Model compared with		1	2	3
Δ*χ*^2^		14.39***	9.30**	69.19***
Δdf		1	1	1

Data are expressed as mean (*SE*).

Time = time in weeks; Group: 0 = treatment-as-usual waiting list, 1 = dialectical behavioural therapy for post-traumatic stress disorder; Index trauma: 0 = multiple traumas, 1 = worst single incident; Δ*χ*^2^, differences in −2*log likelihood between the full model and submodel; Δdf, change between the submodel and full model.

**p ≤ .01, ***p ≤ .001.

### 3.6. Trauma-related and non-specific symptoms in the DBT-PTSD group

Table 5 presents the mean CAPS severity scores for trauma-related symptoms and non-specific symptoms, for the DBT-PTSD group only. At week 24 (t4), for trauma-related symptoms, the mean severity score assessed in relation to the worst single incident was significantly lower compared to the score assessed in relation to multiple traumas: 1.36 (*SD *= 0.86) vs 2.00 (*SD *= 0.92), respectively (*z *= −3.93, *p *< .001, *g *= 0.71). In contrast, for non-specific symptoms, the mean severity scores were virtually the same for both assessments: 1.76 (*SD *= 0.76) for the worst single incident versus 1.74 (*SD *= 0.76) for multiple traumas (*z *= −1.34, *p *= .18, *g *= 0.03). The differences between pre-treatment scores (week 0) and end of study scores (week 24) are shown graphically in Figure 2.

**Table 5.** Mean Clinician-Administered PTSD Scale (CAPS) scores for trauma-related and non-specific symptoms in the dialectical behavioural therapy for post-traumatic stress disorder (DBT-PTSD) group.

		Trauma-related symptoms		Non-specific symptoms	
	*n*	Worst single incident	Multiple traumas	Worst single incident	Multiple traumas
Week 0 (admission, t1)	24	2.84 (0.53)	2.96 (0.47)	2.44 (0.52)	2.44 (0.52)
Week 12 (discharge, t2)	22	1.49 (0.83)	1.93 (0.92)	1.61 (0.70)	1.61 (0.69)
Week 18 (6 week follow-up, t3)	21	1.22 (0.82)	2.18 (0.81)	1.87 (0.73)	1.89 (0.73)
Week 24 (12 week follow-up, t4)	24	1.36 (0.86)	2.00 (0.92)	1.76 (0.76)	1.74 (0.76)
*p^a^* (within-group; t1–t4)		<.001	<.001	.001	<.001
Hedges’ *g* (within-group; t1–t4)		2.04	1.29	1.03	1.06

Data are expressed as mean (*SD*).

^a^ Wilcoxon signed rank test.

## 4. Discussion

This study is the first in which two different definitions of index trauma have been used within the same treatment trial to assess PTSD severity scores and their changes over time. The findings indicate that using different definitions of index trauma in patients who have experienced multiple traumas has an impact on PTSD severity scores. Baseline severity scores were significantly higher when the index trauma included multiple distinct traumatic events compared to when the index trauma was defined as the worst single incident. With respect to the DBT-PTSD group, the definition of index trauma had only a small impact on PTSD severity scores at baseline, but large differences were seen over time, with scores at each post-treatment time-point being higher when assessment was conducted in relation to multiple traumas as compared to the worst single incident. Accordingly, the way in which the index trauma was defined also affected the assessed treatment effect sizes, with less improvement in PTSD symptoms related to multiple traumas compared to PTSD symptoms related to the worst single incident. Dividing PTSD symptoms into those inherently linked to traumatic events (‘trauma-related’) and more general ones (‘non-specific’) revealed that different index trauma definitions were only reflected within the trauma-related symptoms. Taken together, these findings underscore the importance of how the index trauma is defined in patients with multiple traumas.

Our findings confirm and extend existing research on index trauma definitions. When patients in this study were asked on which traumatic event they based their current post-traumatic symptoms, almost all (95%) responded that they were based on more than one traumatic event. This is in line with Karam et al. (2014), who reported that some participants associated their current post-traumatic symptoms with more than one traumatic event; however, our findings indicate that this seems to be the rule rather than the exception, at least in some patient populations.

There are some limitations to this study. First, DBT-PTSD is a trauma-focused treatment that includes interventions from DBT as well as trauma-focused cognitive and exposure-based interventions. While the treatment sessions on trauma-related cognitions, emotion regulation, and radical acceptance of trauma-related and biographical facts focus on all currently distressing traumatic events, the imaginal exposure is conducted in relation to the currently most distressing traumatic incident. Exposure is usually addressed over approximately seven out of 23 individual sessions. No conclusion can be drawn for other trauma-focused treatments. It might be that treatments using a different approach (e.g. cognitive processing therapy; Resick, Monson, & Chard, 2016) or even exposure-based interventions not focusing exclusively on the worst single incident (e.g. prolonged exposure; Foa et al., 2007; narrative exposure therapy; Schauer, Neuner, & Elbert, 2011; eye movement desensitization and reprocessing; Shapiro, 2018) would be less sensitive to different index trauma definitions. Secondly, while patients reported a mean of 6.4 different types of traumatic events, the index trauma definition that we used for the multiple trauma condition included only up to three distinct traumatic events. Consequently, our assessment of ‘multiple traumas’ probably underestimated the overall post-traumatic symptomatology, which suggests that there may be an even larger difference between the different index trauma definitions. Thirdly, we used the CAPS for DSM-IV (Blake et al., 1995) to assess PTSD. The CAPS-5 (Weathers, Blake et al., 2013) specifically permits the assessment of either the worst incident or multiple but closely related incidents. However, our multiple trauma condition included more traumatic events than the CAPS-5 since the three events were qualitatively distinct traumatic events (e.g. CSA, rape, physical abuse). Finally, while model diagnostics indicated an acceptable fit for the hierarchical linear models used in this study, the fit might be improved by using more general (e.g. polynomial) models. However, as hierarchical linear models are the current standard in the field, the predefined primary strategy for testing (linear models) was used, which allows for a better comparison with the existing literature.

Several of our findings should be relevant for both research and clinical practice. With respect to research, first, it appears to be essential that PTSD studies both report the number of currently distressing traumatic events and provide which index trauma definition is being used. If these information are lacking comparisons of treatment outcomes of different types of trauma-focused therapy may be impeded. Of the studies we found in our literature search, fewer than one-quarter reported a definition. Secondly, the instruments used for assessing the severity of PTSD should be supplemented by a broader assessment that addresses the effects of exposure to multiple traumas. Despite the change in DSM-5 (American Psychiatric Association, 2013), almost all diagnostic instruments for PTSD use narrow definitions of the index trauma. The two most widely used interviews, the CAPS-5 (Weathers, Blake et al., 2013) and the PSSI-5 (Foa, McLean, Zang, Zhong, Rauch et al., 2016), assess post-traumatic symptoms in relation to a worst single incident or multiple but closely related incidents. Similarly, self-report instruments, such as the Posttraumatic Checklist for DSM-5 (PCL-5) (Weathers, Litz et al., 2013) and the PDS for DSM-5 (PDS-5) (Foa, McLean, Zang, Zhong, Powers et al., 2016), consider only symptoms in relation to the most distressing traumatic event. Focusing on just a single event may miss other significant aspects of symptomatology when assessing the patient’s overall status and well-being when evaluating treatment effects. In an extreme case, a woman with a history of both CSA and an adulthood rape may identify the latter as the currently most distressing event, with the consequence that the intrusions related to the CSA would not be taken into account when determining the diagnosis and the severity of PTSD. In psychotherapy research, she may be classified as being in remission if she no longer experiences intrusions with respect to the adulthood event, even though she is still suffering from intrusions, flashbacks, and nightmares related to the childhood abuse.

With respect to clinical practice, it appears to be of importance to account for the cumulative effects of trauma in patients with a history of multiple traumatic events. Almost all patients in this study attributed their current PTSD symptoms to more than one trauma. Even though several exposure-based interventions focus on more than one distressing memory, the usual assumption is that exposure focusing on the most disturbing memory will generalize to other memories so that they too will become less distressing (Foa et al., 2007). However, our data suggest that improvements with respect to the most disturbing trauma may not fully generalize to all traumas. Foa et al. (2007) comment, ‘sometimes, even after processing the most distressing memory, another traumatic experience continues to trigger high levels of distress’ (p. 80). Consequently, it may be important to differentiate between patients with a history of multiple traumatic events who attribute their symptoms to one trauma and patients who attribute them to several events. Our results suggest that for the latter, clinicians should consider targeting more than one traumatic memory.

Issues arising from this research may be summarized as follows: (1) as only DBT-PTSD was examined in this study, further research on index trauma definitions should be conducted on other types of trauma-focused treatment; (2) patients in this study had extremely high rates of trauma and were highly symptomatic, so other patient groups need to be investigated as well; (3) a greater variety of index trauma definitions should be investigated in order to gather further information about generalization effects in exposure-based treatments; (4) future trauma-focused treatment studies should describe trauma histories and their index trauma definition clearly; and (5) assessments of PTSD severity should be supplemented to allow symptom assessment and check for PTSD diagnosis in relation to all relevant traumatic events. These measures would help researchers and clinicians to gain a better understanding of trauma-focused treatments in regard to their overall effectiveness.
