# Supplementary material for: Defining the index trauma in post-traumatic stress disorder patients with multiple trauma exposure: impact on severity scores and treatment effects of using worst single incident versus multiple traumatic events
Source: Eur J Psychotraumatol. 2018 Jul 9;9(1):1486124. doi: 10.1080/20008198.2018.1486124 (PMC6052424; doi:10.1080/20008198.2018.1486124)
Supplement: Supplemental Material [file ZEPT_A_1486124_SM6932.docx]

**Supplementary Material**

*Supplementary Table 1: Index trauma definition in randomized controlled trials evaluating the efficacy of psychological treatments in adults with repeated childhood abuse*

| Study | | |  | Trauma | |  | PTSD outcome measure | |  | Results treatment group^a)^ | | | | |
| --- | --- | --- | --- | --- | --- | --- | --- | --- | --- | --- | --- | --- | --- | --- |
| Author | Intervention^a)^ | Total sample |  | Treatment target | History |  | Instrument | Index trauma definition |  | Intent-to-treat | |  | Completer | |
|  |  |  |  |  |  |  |  |  |  | Mean (SD)  pre post | Effect size^b)^ *g* |  | Mean (SD)  pre post | Effect size^b)^ *g* |
| Bohus  et al. (2013) | 12 weeks of inpatient **Dialectical Behavior Therapy for Posttraumatic Stress Disorder (DBT-PTSD)** vs. TAU-WL | 74 females with PTSD related to CSA |  | CSA | CSA 100% |  | CAPS | “CSA event currently causing highest distress” |  | 87.9 (14.2)  60.3 (26.8)  *n* = 36 | 1.27 |  | 87.9 (14.2)  57.2 (25.5)  *n* = 29 | 1.47 |
| Bradley & Folling-stad (2003) | 18 sessions of **Supportive Group Therapy (DBT skills and writing assignments)** vs. No contact group | 49 incarcerated females with a history of childhood abuse |  | Non-trauma-focused | CSA 100%  CPA 71%  ASA 83%  APA 90% |  | TSI; subscale intrusive experiences | Not reported |  | - | - |  | 66.5 (8.8)  56.6 (11.1)  *n* = 13 | 0.96 |
| Chard (2005) | 17 sessions of group therapy and 10 sessions of individual therapy **Cognitive Processing Therapy for Sexual Abuse (CPT-SA)** vs. Minimal Attention WL | 71 females with PTSD related to CSA |  | CSA | CSA 100% |  | CAPS | Not reported |  | - | - |  | 65.5 (26.4)   9.0 (11.0)  *n* = 28 | 2.75 |
| Cloitre  et al. (2002) | 16 individual sessions of **Skills Training (STAIR) + Exposure** vs. Minimal Attention WL | 58 females with PTSD related to CSA and/or CPA |  | CSA and/or CPA | CSA 87% CPA 61% |  | CAPS | Not reported |  | - | - |  | 69.0 (16.3)  31.0 (25.2)  *n* = 22 | 1.76 |
| Cloitre  et al. (2010) | 16 individual sessions of **Skills Training (STAIR) + Exposure** vs. Skills Training + Support vs. Support + Exposure | 104 females with PTSD related to CSA and/or CPA |  | CSA and/or CPA | CSA 89%  CPA 81% ASA 55% APA 22% |  | CAPS | Not reported |  | 63.1 (18.1)  32.7 (19.4)  *n* = 33 | 1.59 |  | - | - |
| Dorrepaal et al. (2012) | 20 sessions of **Stabilizing Group Treatment + TAU** vs. TAU | 71 females with PTSD related to CSA or CPA |  | Non-trauma-focused | CSA 94%  CPA 63%  ASA 48%  APA 43% |  | DTS | Not reported |  | 89.8 (20.3)  69.6 (27.4)  *n* = 38 | 0.83 |  | 91.4 (21.8)  66.7 (29.4)  *n* = 31 | 0.94 |
| Edmond et al. (1999) | 6 individual sessions of **EMDR** vs. Routine Treatment vs. WL | 59 females with a history of CSA |  | “most troubling issue”, “specific memory” | CSA 100% CPA 58% Adulthood trauma 66% |  | IES | Not reported |  | 38.7 (16.4)  14.1 (15.9)  *n* = 20 | 1.49 |  | - | - |
| Jung & Steil (2013) | 2 sessions of **Cognitive Restructuring and Imagery Modification (CRIM)** vs. WL | 34 females with PTSD related to CSA and the feeling of being contaminated |  | Non-trauma-focused | CSA 100% |  | CAPS | Not reported |  | - | - |  | 80.6 (20.2)  60.9 (23.3)  *n* = 14 | 0.88 |
| Krupnick et al. (2008) | 16 group sessions of **Interpersonal Therapy (IPT)** vs. WL | 48 females with PTSD after an interpersonal trauma |  | Non-trauma-focused | CSA 96% CPA 96% |  | CAPS | Not reported |  | 65.2 (20.9)  40.6 (16.9)  *n* = 24 | 1.27 |  | - | - |
| McDonagh et al. (2005) | 14 individual sessions of **Cognitive-Behavioral Therapy (CBT)** vs. Present-Centered Therapy (PCT) vs. WL | 74 females with PTSD related to CSA |  | CSA | CSA 100% CPA 80% ASA 51% APA 63% |  | CAPS | “a CSA experience” |  | 69.9 (16.8)  53.1 (28.8)  *n* = 29 | 0.70 |  | 67.1 (18.4)  38.5 (27.7)  *n* = 17 | 1.19 |
| Paivio et al. (2010) | 18 individual sessions of **Emotion-focused Therapy with imaginal confrontation** vs. Emotion-focused Therapy with empathic exploration | 56 females and males with histories of childhood maltreatment |  | CSA 56% CPA 13% Emotional abuse 22% Neglect 9% | multiple types of childhood maltreat-ment 69% |  | PSS-I | Not reported |  | - | - |  | 23.2 (11.4)  10.4 (10.3)  *n* = 20 | 1.16 |
| Resick  et al.  (2008) | 12 hours of individual therapy (7 – 12 sessions) of **Cognitive Processing Therapy (CPT)** vs. Cognitive Therapy only (CPT-C) vs. Written Accounts (WA) | 150 females with PTSD after interpersonal violence |  | CSA 38% CPA 7% ASA 31% APA 23% | CSA 78 % APA 84% ASA 81% |  | CAPS | “worst event” |  | 70.2 (15.5)  34.7 (27.6)  *n* = 42 | 1.62 |  | - | - |
| Scheck et al. (1998) | 2 individual sessions of **EMDR** vs. Active Listening Control | 67 females with a traumatic memory and dysfunctional behavior |  | Not reported | CSA and/or CPA 90% |  | IES | Not reported |  | - | - |  | 48.01 (11.8)  23.4 (18.4)  *n* = 28 | 1.57 |
| Sikkema et al. (2007) | 15 group sessions of **HIV and Trauma Coping Intervention** vs. HIV Support Group Intervention vs. WL | 253 females and males with HIV/AIDS and a history of CSA |  | CSA 100% | CSA 100% ASA 53% |  | IES-intrusion subscale | “in reference to their sexually traumatic experience” |  | - | - |  | 16.8 (9.5)  12.5 (9.3)  *n* = 73 | 0.45 |
| van der Kolk et al. (2007) | 8 individual sessions of **EMDR** vs. Fluoxetine vs. Pill Placebo | Subgroup of 45 females and males with PTSD related to childhood trauma |  | “Memories associated with the primary trauma”  CSA 73% CPA 9% | Not reported |  | CAPS | Not reported |  | - | - |  | 73.5 (12.9)  38.4 (20.7)  *n* = 11 | 2.35 |
| Zlotnick et al. (1997) | 15 group sessions of **Affect-management Group** Treatment vs. WL | 48 females with PTSD related to CSA |  | Non-trauma-focused | CSA 100% ASA 77% |  | DTS | Not reported |  | - | - |  | 66.9 (22.0)  45.8 (34.1)  *n* = 16 | 1.11 |
| Zlotnick et al. (2009) | 24 group sessions of **Seeking Safety plus TAU** vs. TAU | 49 incarcerated females with PTSD and substance abuse disorder |  | Non-trauma-focused | Sexual abuse 94%  Physical abuse 90% |  | CAPS | Not reported |  | - | - |  | 69.4 (16.7)  57.0 (23.7)  *n* = 27 | 0.60 |

*Note.*: ASA = adult sexual assault, APA = adult physical assault, CAPS = Clinician-Administered PTSD Scale (Blake et al., 1995), CSA = child sexual abuse, CPA = child physical abuse, DTS = Davidson Trauma Scale (Davidson et al., 1997), IES = Impact of Event Scale (Horowitz et al., 1979), PTSD = posttraumatic stress disorder, PSS-I = PTSD Symptom Scale Interview (Foa et al., 1993), TAU = Treatment as usual, TSI = Trauma Symptom Inventory (Briere, 1995), WL = Wait list.
^a)^ Outcomes reported for the treatment group marked in bold, pre- and post-treatment.
^b)^ Estimator of effect size Hedges’ g was based on the pooled standard deviation and included correction to adjust overestimation of the population effect size in small samples (Hedges & Olkin, 1985, p. 81).

Blake, D. D., Weathers, F. W., Nagy, L. M., Kaloupek, D. G., Gusman, F. D., Charney, D. S., & Keane, T. M. (1995). The development of a Clinician-Administered PTSD Scale*. Journal of Traumatic Stress, 8,* 75-90.

Bohus, M., Dyer, A. S., Priebe, K., Krüger, A., Kleindienst, N., Schmahl, C., . . . Steil, R. (2013). Dialectical behaviour therapy for post-traumatic stress disorder after childhood sexual abuse in patients with and without borderline personality disorder: A randomised controlled trial. *Psychotherapy and Psychosomatics, 82,* 221-233.

Bradley, R. G., & Follingstad, D. R. (2003). Group therapy for incarcerated women who experienced interpersonal violence: A pilot study. *Journal of Traumatic Stress, 16,* 337-340.

Briere, J., Elliott, D. M., Harris, K., & Cotman, A. (1995). Trauma symptom inventory psychometrics and association with childhood and adult victimization in clinical samples. *Journal of Interpersonal Violence, 10,* 387-401.

Chard, K. M. (2005). An evaluation of cognitive processing therapy for the treatment of posttraumatic stress disorder related to childhood sexual abuse. *Journal of Consulting and Clinical Psychology, 73*, 965-971.

Cloitre, M., Koenen, K. C., Cohen, L. R., & Han, H. (2002). Skills training in affective and interpersonal regulation followed by exposure: A phase-based treatment for PTSD related to childhood abuse. *Journal of Consulting and Clinical Psychology, 70,* 1067-1074.

Cloitre, M., Stovall-McClough, C. K., Nooner, K., Zorbas, P., Cherry, S., Jackson, C. L., . . . Petkova, E. (2010). Treatment for PTSD related to childhood abuse: A randomized controlled trial. *American Journal of Psychiatry, 167,* 915-924.

Davidson, J. R., Book, S. W., Colket, J. T., Tupler, L. A., Roth, S., David, D., ... & Davison, R. M. (1997). Assessment of a new self-rating scale for post-traumatic stress disorder. *Psychological Medicine, 27,* 153-160.

Dorrepaal, E., Thomaes, K., Smit, J. H., Van Balkom, A. J., Veltman, D. J., Hoogendoorn, A. W., & Draijer, N. (2012). Stabilizing group treatment for complex posttraumatic stress disorder related to child abuse based on psychoeducation and cognitive behavioural therapy: A multisite randomized controlled trial. *Psychotherapy and Psychosomatics, 81,* 217-225.

Edmond, T., Rubin, A., & Wambach, K. G. (1999). The effectiveness of EMDR with adult female survivors of childhood sexual abuse. *Social Work Research, 23,* 103-116.

Feske, U. (2008). Treating low-income and minority women with posttraumatic stress disorder: a pilot study comparing prolonged exposure and treatment as usual conducted by community therapists. *Journal of Interpersonal Violence, 23*, 1027-1040.

Foa, E. B., Riggs, D. S., Dancu, C. V., & Rothbaum, B. O. (1993). Reliability and validity of a brief instrument for assessing post‐traumatic stress disorder. *Journal of Traumatic Stress, 6,* 459-473.

Hedges, L. V., & Olkin, I. (1985). *Statistical methods for meta-analysis*. Orlando: Academic Press.

Horowitz, M., Wilner, N., & Alvarez, W. (1979). Impact of Event Scale: a measure of subjective stress. *Psychosomatic Medicine, 41,* 209-218.

Jung, K., & Steil, R. (2013). A randomized controlled trial on cognitive restructuring and imagery modification to reduce the feeling of being contaminated in adult survivors of childhood sexual abuse suffering from posttraumatic stress disorder. *Psychotherapy and Psychosomatics, 82,* 213-220

Krupnick, J. L., Green, B. L., Stockton, P., Miranda, J., Krause, E., & Mete, M. (2008). Group interpersonal psychotherapy for low-income women with posttraumatic stress disorder. *Psychotherapy Research, 18,* 497-507.

McDonagh, A., Friedman, M., McHugo, G., Ford, J., Sengupta, A., Mueser, K., . . . Descamps, M. (2005). Randomized trial of cognitive-behavioral therapy for chronic posttraumatic stress disorder in adult female survivors of childhood sexual abuse. *Journal of Consulting & Clinical Psychology, 73,* 515-524

Paivio, S. C., Jarry, J. L., Chagigiorgis, H., Hall, I., & Ralston, M. (2010). Efficacy of two versions of emotion-focused therapy for resolving child abuse trauma. *Psychotherapy Research, 20,* 353-366.

Resick, P. A., Galovski, T. E., Uhlmansiek, M. O. B., Scher, C. D., Clum, G. A., & Young-Xu, Y. (2008). A randomized clinical trial to dismantle components of cognitive processing therapy for posttraumatic stress disorder in female victims of interpersonal violence. *Journal of Consulting and Clinical Psychology, 76,* 243-258.

Scheck, M. M., Schaeffer, J. A., & Gillette, C. (1998). Brief psychological intervention with traumatized young women: The efficacy of eye movement desensitization and reprocessing. *Journal of Traumatic Stress, 11,* 25-44.

Sikkema, K. J., Hansen, N. B., Kochman, A., Tarakeshwar, N., Neufeld, S., Meade, C. S., & Fox, A. M. (2007). Outcomes from a group intervention for coping with HIV/AIDS and childhood sexual abuse: reductions in traumatic stress. *AIDS and Behavior, 11,* 49-60.

van der Kolk, B. A., Spinazzola, J., Blaustein, M. E., Hopper, J. W., Hopper, E. K., Korn, D. L., & Simpson, W. B. (2007). A randomized clinical trial of eye movement desensitization and reprocessing (EMDR), fluoxetine, and pill placebo in the treatment of posttraumatic stress disorder: treatment effects and long-term maintenance. *Journal of Clinical Psychiatry, 68,* 37-46.

Zlotnick, C., Johnson, J., & Najavits, L. M. (2009). Randomized controlled pilot study of cognitive-behavioral therapy in a sample of incarcerated women with substance use disorder and PTSD. *Behavior Therapy, 40,* 325-336.

Zlotnick, C., Shea, T. M., Rosen, K., Simpson, E., Mulrenin, K., Begin, A., & Pearlstein, T. (1997). An affect‐management group for women with posttraumatic stress disorder and histories of childhood sexual abuse. *Journal of Traumatic Stress, 10,* 425-436.

Assessed for Eligibility *N*=176

Randomized *N*=82

Did not receive allocated treatment:

Refused treatment *n*= 4

Protocol violation *n*= 3

Received allocated treatment *n*=36 / 29

Both CAPS assessments 24

Treatment completers *n*=34 / 27

Treatment dropouts *n*= 2 / 2

Did not receive allocated treatment:

Refused treatment *n*= 1

Received allocated treatment *n*=38 / 29

Both CAPS assessments 23

12 weeks after admission (t2)

Assessed *n*=34 / 27

Both CAPS assessments 22

Refused assessment *n*= 2 / 2

18 weeks after admission (t3)

Assessed *n*=28 / 22

Both CAPS assessments 21

Refused assessment *n*= 8 / 7

24 weeks after admission (t4)

Assessed *n*=29 / 24

Both CAPS assessments 24

Refused assessment *n*= 7 / 5

12 weeks after randomization (t2)

Assessed *n*=34 / 25

Both CAPS assessments 22

Refused assessment *n*= 3 / 3

Not reached *n*= 1 / 1

18 weeks after randomization (t3)

Assessed *n*=32 / 23

Both CAPS assessments 22

Refused assessment *n*= 5 / 5

Not reached *n*= 1 / 1

24 weeks after randomization (t4)

Assessed *n*=33 / 25

Both CAPS assessments 25

Refused assessment *n*= 4 / 3 Not reached *n*= 1 / 1

Data used in HLM analysis: *n*=36 / 29

Data used in HLM analysis: *n*=38 / 29

Randomized to DBT-PTSD (t1) *n*=43

Randomized to TAU-WL (t1) *n*=39

Excluded *n*=94

Did not complete assessment *n*=13

Did not meet all inclusion criteria *n*=30

Met at least 1 exclusion criterion *n*=18

Chose not to participate *n*=33

*Supplementary Figure 1.* Patient flow through enrolment, allocation, follow-up, and analysis. Where applicable, numbers for both the main study and for just the subsample used in the current study are displayed (main study/subsample).

Supplementary Table 2: *Traumatic event types assembled out of Posttraumatic Diagnostic Scale (PDS) and Life Events Checklist (LEC)*

|  | Description of traumatic event type | |  |
| --- | --- | --- | --- |
| Count | PDS ([Foa, 1995](#_ENREF_17)) | LEC ([Blake et al., 1995](#_ENREF_6)) | Comment |
| 4 | Serious accident, fire or explosion | Fire or explosion | Up to 4 counts, if more than one question in the LEC was ticked off |
|  |  | Traffic accident |  |
|  |  | Serious accident at work, home, or during recreational activity |  |
|  |  | Exposure to toxic substance |  |
| 1 | Natural disaster | Natural disaster |  |
| 1 | Non-sexual assault by a family member or someone you know | Physical assault  Assault with a weapon |  |
| 1 | Non-sexual assault by a stranger |  |  |
| 1 | Sexual assault by a family member | Sexual assault |  |
| 1 | Sexual assault by a stranger |  |  |
| 1 |  | Other unwanted or uncomfortable sexual experience |  |
| 1 | Military or combat war zone | Combat or exposure to a war zone |  |
| 1 | Sexual contact when younger than 18 with someone who was 5 or more years older |  |  |
| 1 | Imprisonment | Captivity |  |
| 1 | Torture |  |  |
| 1 | Life-threatening illness | Life-threatening illness or injury |  |
| 1 |  | Other unwanted or uncomfortable sexual experience |  |
| 1 |  | Severe human suffering |  |
| 1 |  | Sudden, violent death |  |
| 1 |  | Sudden unexpected death of someone close to you |  |
| 1 |  | Serious injury, harm, or death to someone else that was caused by you |  |
| 1 | Other traumatic event | Any other very stressful event or experience | Only if traumatic event was not mentioned before |
| 21 |  |  | Different event types, total score |

*Supplementary Table 3: Mean CAPS scores based on DSM- IV for Criteria B, C and D*

|  |  |  | DBT-PTSD | | |  | TAU-WL | | |
| --- | --- | --- | --- | --- | --- | --- | --- | --- | --- |
|  |  |  | Worst single incident |  | Multiple traumas |  | Worst single incident |  | Multiple traumas |
|  |  |  |  |  | Criterion B | | |  |  |
|  | Week 0 (admission, t1) |  | 2.92 (0.76) |  | 3.05 (0.61) |  | 2.64 (0.90) |  | 2.93 (0.89) |
|  | Week 12 (discharge, t2) |  | 1.83 (1.00) |  | 2.25 (1.05) |  | 2.72 (0.73) |  | 2.96 (0.61) |
|  | Week 18 (6-week follow-up, t3) |  | 1.34 (0.95) |  | 2.40 (0.86) |  | 2.71 (0.76) |  | 3.04 (0.62) |
|  | Week 24 (12-week follow-up, t4) |  | 1.58 (1.02) |  | 2.27 (1.09) |  | 2.57 (0.84) |  | 2.60 (0.86) |
|  | Hedges’ *g* (within-group; t1-t4) |  | 1.47 |  | 0.87 |  | 0.08 |  | 0.37 |
|  |  |  |  |  | Criterion C | | |  |  |
|  | Week 0 (admission, t1) |  | 2.54 (0.56) |  | 2.59 (0.58) |  | 2.51 (0.43) |  | 2.61 (0.47) |
|  | Week 12 (discharge, t2) |  | 1.20 (0.66) |  | 1.41 (0.69) |  | 2.46 (0.65) |  | 2.45 (0.66) |
|  | Week 18 (6-week follow-up, t3) |  | 1.36 (0.74) |  | 1.72 (0.82) |  | 2.46 (0.62) |  | 2.50 (0.64) |
|  | Week 24 (12-week follow-up, t4) |  | 1.40 (0.81) |  | 1.62 (0.84) |  | 2.41 (0.61) |  | 2.47 (0.60) |
|  | Hedges’ *g* (within-group; t1-t4) |  | 1.61 |  | 1.32 |  | 0.19 |  | 0.25 |
|  |  |  |  |  | Criterion D | | |  |  |
|  | Week 0 (admission, t1) |  | 2.45 (0.62) |  | 2.45 (0.62) |  | 2.63 (0.41) |  | 2.63 (0.41) |
|  | Week 12 (discharge, t2) |  | 1.76 (0.81) |  | 1.76 (0.82) |  | 2.68 (0.41) |  | 2.68 (0.40) |
|  | Week 18 (6-week follow-up, t3) |  | 2.07 (0.74) |  | 2.08 (0.74) |  | 2.63 (0.67) |  | 2.63 (0.67) |
|  | Week 24 (12-week follow-up, t4) |  | 1.80 (0.79) |  | 1.81 (0.79) |  | 2.56 (0.59) |  | 2.56 (0.59) |
|  | Hedges’ *g* (within-group; t1-t4) |  | 0.90 |  | 0.89 |  | 0.13 |  | 0.13 |

*Note*. Data are expressed as means. Standard deviations are in parentheses. ^a^Participants were only included when CAPS scores were available in relation to the worst single incident and in relation to multiple traumas. This resulted in *n*=24 for t1, *n*=22 for t2, *n*=21 for t3 and *n*=24 for t4 in the DBT-PTSD group, and in *n*=23 for t1, *n*=22 for t2, *n*=22 for t3, and *n*=25 for t4 in the TAU-WL group.
